# Provision of peer specialist services in VA patient aligned care teams: protocol for testing a cluster randomized implementation trial

**DOI:** 10.1186/s13012-017-0587-7

**Published:** 2017-05-02

**Authors:** Matthew Chinman, Karin Daniels, Jeff Smith, Sharon McCarthy, Deborah Medoff, Amanda Peeples, Richard Goldberg

**Affiliations:** 1 VISN 4 Mental Illness Research, Education and Clinical Center, VA Pittsburgh, Pittsburgh, PA USA; 2 Center for Health Equity Research and Promotion, VA Pittsburgh, Pittsburgh, PA USA; 3 RAND Corporation, Pittsburgh, PA USA; 4 Central Arkansas Veterans Healthcare System, HSR&D and Mental Health Quality Enhancement Research Initiative (QUERI), Little Rock, AR USA; 5 VISN 5 Mental Illness Research, Education and Clinical Center (MIRECC), Baltimore, MD USA; 6 Division of Psychiatric Services Research, Department of Psychiatry, University of Maryland, School of Medicine, Baltimore, MD USA; 7 VA Pittsburgh Healthcare System, Research Office Building (151R), University Drive C, Pittsburgh, PA 15240 USA

**Keywords:** Implementation, Facilitation, Peer specialist

## Abstract

**Background:**

Over 1100 Veterans work in the Veterans Health Administration (VHA) as peer specialists (PSs). PSs are Veterans with formal training who provide support to other Veterans with similar diagnoses, primarily in mental health settings. A White House Executive Action mandated the pilot reassignment of VHA PSs from mental health to 25 primary care Patient Aligned Care Teams (PACT) in order to broaden the provision of wellness services that can address many chronic illnesses. An evaluation of this initiative was undertaken to assess the impact of outside assistance on the deployment of PS in PACT, as implementation support is often needed to prevent challenges commonly experienced when first deploying PSs in VHA settings. We present the protocol for this cluster-randomized hybrid type II trial to test the impact of standard implementation (receive minimal assistance) vs. facilitated implementation (receive outside assistance) on the deployment of VHA PSs in PACT.

**Methods:**

A VHA Office of Mental Health Services work group is recruiting 25 Veterans Affairs Medical Centers to reassign a mental health PSs to provide wellness-oriented care in PACT. Sites in three successive cohorts (*n* = 8, 8, 9) beginning over 6-month blocks will be matched and randomized to either standard or facilitated implementation. In facilitated implementation, an outside expert works with site stakeholders through a site visit, regular calls, and performance data to guide the planning and address challenges. Standard implementation sites will receive a webinar and access the Office of Mental Health Services work group. The two conditions will be compared on PS workload data, fidelity to the PS model of service delivery, team functioning, and Veteran measures of activation, satisfaction, and functioning. Qualitative interviews will collect information on implementation barriers and facilitators.

**Discussion:**

This evaluation will provide critical data to guide administrators and VHA policy makers on future deployment of PSs, as their role has been expanding beyond mental health. In addition, development of novel implementation strategies (facilitation tailored to PSs) and the use of new tools (peer fidelity) can be models for monitoring and supporting deployment of PSs throughout VHA.

**Trial registration:**

ClinicalTrials.gov, NCT02732600 (URL:https://clinicaltrials.gov/ct2/show/NCT02732600)

## Background

Persons with mental illness, particularly Veterans, have higher rates of common diseases, e.g., cardiovascular, lung, and digestive [1–12], and worse health outcomes associated with those conditions compared to the general population. Those with mental illness have higher risk for certain lifestyle choices (e.g., obesity, physical inactivity, smoking, and drug use) that contribute to chronic medical illness [13–23], and have a higher mortality rate than the general population as a result [2, 3, 13, 14, 19]. These poor outcomes increase healthcare spending, as 75% of the Veterans Health Administration (VHA) healthcare dollars are spent on these chronic diseases [5].

A key driver for poor outcomes is that those with mental illness often have low “patient activation,” or the knowledge, skill, efficacy, and beliefs for managing personal health and healthcare [24]. For example, they may not verbalize concerns, [4, 14, 25, 26] or they may deny illness, [27] be reluctant to see non-mental health providers [28], accept medical care that is inadequate, [29] or have difficulty accessing care in systems that are often poorly coordinated [30]. Thus, a key area for intervention is to increase the activation of Veterans with mental illness receiving services in primary care. Research shows that more “activated” individuals engage more in self-management (e.g., medication adherence, diet, exercise), disease prevention (e.g., screenings), and health-information seeking [31] and as a result, have better health, functioning, quality of life, and higher satisfaction with their care [32, 33].

To improve services for Veterans, VHA has developed new primary care structures such as Patient Aligned Care Teams (PACT; [34]) to serve as “medical homes” that provide coordinated, comprehensive outpatient care. VHA PACT, first established in 2010, provide outpatient primary care services through patient-centered medical “teamlets”, each of which coordinates comprehensive care for approximately 1200 Veterans [34]. PACT teamlets typically include a primary care physician, a care manager (RN), a medical assistant or LPN, and an administrative clerk. However, even with PACT, Veterans with mental illnesses often receive disparately poor care because of low patient activation which is needed to ensure proper health and use of healthcare [24].

There is emerging evidence that peer specialists (PSs)—individuals with mental illness hired and trained to serve Veterans in specialty mental health clinics based upon their lived experiences [35]—could be a viable option to provide basic coaching to improve patient activation and the health of those with mental illness. PSs are Veteran employees with significant recovery from serious mental illness or substance abuse disorders, who are then trained to provide ongoing support to other Veterans with similar disorders. Their role is unique in that they draw upon their own recovery experience to inform their support of Veterans. The shared experience of military service also fosters trust between PS and the Veterans with whom they serve. The specific roles filled by PS beyond this basic foundation are varied and include facilitating groups, role modeling, providing outreach and support, teaching coping skills, case management and liaison between Veteran and mental health system. While not conclusive, randomized and quasi-experimental trials have shown that those mental illnesses who receive PS services benefit across a wide range of outcomes [35].

Recently, the PS role has been extended to providing wellness-oriented, health services. Druss [36] used PSs in an adaptation of Lorig’s Chronic Disease Self-Management Program (CDSMP), which originally used community lay leaders to teach patients with diverse chronic diseases self-management skills. In a small randomized trial (*n* = 80) involving PSs, intervention participants had significantly greater improvement in patient activation than those in usual care. Participants also experienced improvements in physical health-related quality of life, physical activity, and medication adherence similar to those in Lorig’s work in the general population. Goldberg and colleagues [37] evaluated another adaptation of CDSMP called Living Well, which also used PSs, and found significant improvements in functioning, self-efficacy, patient activation, and self-management.

The use of PSs in a primary care setting is still relatively new. For example, while almost 1100 PSs are working in VHA mental healthcare settings, only a few have worked in PACT. In August 2014, the White House issued an Executive Action mandating that 25 Veterans Affairs Medical Centers (VAMCs), for the first time, pilot the use of PSs in their PACT to provide health coaching [38]. Health coaching is a patient-centered approach that includes assessing needs, developing concrete goals and a plan to reach those goals, and skill building. Providers partner with patients to identify health behavior changes and create action steps and follow-up plans to make those changes. Given the “here and now” emphasis of goal planning, services are meant to be brief, between one and eight sessions. Sites have been offered to have their PSs trained in health coaching by VHA at no cost. While no additional funds were made available, VA Central Office invited all VAMCs to move between 0.2 and 2.0 FTE of existing PSs to PACT at their site to address the executive action.

Over and above the VA Central Office invitation, research shows that proactive strategies are needed to facilitate successful adoption of new interventions [39], and that implementation support is especially needed to prevent challenges commonly experienced when first employing PSs [40–49]. Hence, in partnership with various VHA leaders, VA’s Quality Enhancement Research Initiative (QUERI) provided funding for a cluster randomized trial comparing 13 VAMCs placing PSs in their PACT with minimal assistance to 12 VAMCs placing PSs in their PACT with outside implementation assistance. This paper presents the protocol for the trial.

## Methods

### Overview

This study is a cluster-randomized hybrid type II trial assessing the impact of facilitation implementation support on PS services in PACT over and above standard implementation of PS. As is common in hybrid type II designs [50], the project has a dual emphasis on the assessment of both Veteran-level outcomes and uptake of PS services. The clusters to be randomized are 25 PACT—which include their PSs, providers, and Veterans—who will be randomly assigned to a study condition (standard implementation vs. facilitated implementation). “Standard implementation” is the treatment as usual condition, in which sites receive minimal assistance typical of large mandated roll-outs in VHA. This approach to implementation will include written guidance (a toolkit on how to hire and integrate PSs into a clinical setting), a 1-h webinar on integrating mental health and primary care, and the option to call VHA Central Office staff overseeing the pilot for ad hoc consultation. Sites randomized to the “facilitated implementation” condition will receive the same resources augmented by additional proactive assistance.

### Facilitated implementation model and strategy

Proactive support for each site randomized to the facilitated implementation condition will be provided for 1 year by one of three doctorate-level psychologists trained in the facilitation model called Integrated Promoting Action Research on Implementation in Health Services (i-PARIHS) [51–56]. According to i-PARIHS, successful implementation of an innovation (in this case PS on PACT) is the function of (1) characteristics of implementation *recipients* (such as their beliefs, skills, and resources); (2) the existence and appraisal of the *evidence* for an innovation by those recipients; (3) characteristics of the immediate work setting or *inner context* (e.g., leadership support, culture, organizational priorities) and the wider health system or *outer context* (e.g., policy drivers and priorities, incentives and mandates); and (4) the process by which outside individuals proactively work with a site to help them adopt and implement an innovation, i.e., *facilitation*, described below [51].

Facilitators will engage in several strategies, tailored to each location [57], including identifying and engaging key stakeholders, opinion leaders and clinical champions at all organizational levels; identifying problems and finding solutions, providing assistance with technical issues, developing information exchange networks, providing evidence, marketing the use of PSs to PACT staff, staff training, formative evaluation, and role modeling [50, 55, 57–59]. In this project, the facilitation is being adapted to also incorporate lessons learned about the implementation of PSs [24, 57]. The above strategies are implemented in two phases, pre-implementation and implementation, further described in Table 1.

**Table 1:** Planned external facilitator roles

Pre-implementation	*Conduct site visit to assist PC stakeholders in developing a local implementation plan (LIP)* ♦ Clarify purpose/role of facilitation staff; share organizational assessment data; set expectations ♦ Assess and engage facility stakeholders who will be impacted by the implementation ♦ Educate PACT on implementation strategies and/or PS evidence (e.g., VA’s PS toolkit, https://www.mirecc.va.gov/visn4/peer_specialist_toolkit.asp) ♦ Assist PACT in developing goals for assessing progress in achieving LIP using an existing facilitation worksheet (critical tasks, persons responsible, PACT needs) modified for this project ♦ Address needs for local customization prior to implementation
Implementation	*Maintain a supportive relationship with stakeholders via multiple means* ♦ Biweekly calls to discuss status of implementation and problem-solving (see below) as needed ♦ Monthly calls to include all facilitation sites (within each cohort) to facilitate information sharing (called “Learning collaboratives”) ♦ Accessibility to stakeholders by telephone/email for additional support or consultation as needed
	*Problem identification and resolution* ♦ Monitor and provide feedback on progress in achieving implementation goals/milestones ♦ Aid problem-solving by leveraging local resources, sharing solutions, or identifying VA resources ♦ Monitor use and impact of identified solutions for problems/barriers

The three facilitators are located in Pittsburgh, PA, and thus most of the facilitation will take place via conference call. Prior to initiating implementation activities at each site (pre-implementation phase), the assigned external facilitator will interview designated internal champions and other key PACT team members (*recipients*) to assess the *inner and outer context*, specifically focusing on any existing barriers and facilitators to implementation. Based on these interviews, the facilitation team will develop an implementation checklist [60], outlining key tasks that will be important for each site to address. In-person site visits will then be conducted to (a) provide evidence and education for the provision of PS services in PACT to leadership, providers and administration (*innovation)*; (b) partner with the PACT team (*recipients*) to develop an implementation plan tailored to site-level needs (*inner context*), and (c) develop a plan to monitor implementation. This monitoring (implementation phase) will include twice monthly meetings to help facilities implement and refine their plans, review goals and assess and address barriers. The process will be further supported through the iterative feedback of data gleaned through the capture of PS workload data through the VHA electronic medical record. In addition, two separate monthly “learning collaborative” conference calls will be held; one for PSs and one for PSs supervisors. Facilitated by a consultant expert in PSs, these calls will involve site staff meeting together to review implementation progress, share ideas and lessons learned, and provide support.

### Site recruitment and randomization

A convenience sample of 25 PACT sites will be recruited through a work group of VHA leaders led by the VA’s National Director of Peer Support and National Director of Integrated Services. To participate, sites pledge to dedicate one or more PSs to PACT for a total of at least 10 h a week. In a brief application, interested sites specify a local champion, the number and make-up of the target population the PS will serve, and specific objectives to be achieved. Site leadership (site director, mental health and primary care leads) must indicate their support. Recruited sites will be divided into three cohorts (*n* = 8, 8, 9). Each cohort will begin over three successive 6-month blocks. The sites in each cohort will be placed into matched pairs based on the following variables: number of PSs deployed at a site, number of hours each PS would provide in PACT, the status of PS assignment to PACT (currently deployed or anticipating deployment to PACT), and employment status (currently hired or within the hiring process). A statistician with no other direct involvement in the project will use a computerized random number generator to assign sites within each pair to 1 year of standard implementation (*n* = 13) or facilitated implementation (*n* = 12). Blinding to assignment will not be possible because it will be well known who receives the facilitation. The project is considered quality improvement by the VA; thus, individual consent will not be sought. However, individual Veterans and staff will be asked for their assent after receiving information about the project.

### Data collection and analysis

Data collection and analysis is guided by the RE-AIM evaluation framework, which is often used as a complement to i-PARIHS in implementation research [52, 61–63]. RE-AIM specifies five domains to judge successful impact of an intervention: *R*each (the spread of the intervention to targeted individuals), *E*ffectiveness (the ability of the intervention to improve outcomes), *A*doption (the integration of the intervention into current practice), *I*mplementation (the fidelity with which the intervention is delivered), and *M*aintenance (the sustainability of the intervention over time). *R*each, *A*doption, and *I*mplementation will be assessed with a variety of context and service delivery measures. The *E*ffectiveness domain will be assessed with Veteran-level measures. The final domain, *M*aintenance, is assessed over time, by repeating the context, service delivery, and Veteran-level measures. Blinded primary data collection will not be possible because the measures are self-report and respondents will know their study condition.

#### Context measures

The Organizational Readiness for Change (ORC; [64]) measures organizational functioning and climate, consistent with the inner and outer context factors of the i-PARIHS framework, and was recently adapted for use with VA providers treating those with mental illnesses [65]. It assesses the following domains: motivation for change, resources, staff attributes, organizational climate, and training exposure and utilization. To minimize participant burden as has been done in other VA implementation studies [65], only 10 of 23 ORC subscales (59 items total) will be administered. These subscales were drawn from the domains of motivation for change (scales including: program needs, training needs, and pressures for change) resources (staffing) and organizational climate (mission, cohesion, autonomy, communication, stress, and change). The 10 ORC subscales have Cronbach’s alpha ranging from 0.57 to 0.87 [64], and in a recent systematic review of instruments for measuring organizational readiness for change, the ORC was rated as the most psychometrically sound based on the Standards for Educational and Psychological Testing, with evidence of multiple forms of validity and reliability [66]. Via an email link to an online survey portal, the ORC will be administered only at baseline to all site staff involved with the Peers in PACT project in any capacity.

The Team Development Measure (TDM; [67]) is a 10-min, 31-item survey designed to measure and promote quality improvement in team-based healthcare settings. The TDM evaluates team development across four key elements: cohesiveness, communication, role clarity, and goals and means clarity. In psychometric testing of the TDM in 145 healthcare teams ranging from 3 to 30 members, the measure had a Cronbach’s alpha of 0.97 and a Rasch/IRT: person reliability of 0.96 [67]. The TDM will be completed by the subset of PACT staff who work directly with the PS. While this will typically include three to four individuals per team, the number will vary as each site will define the scope of the PS role differently (i.e., collaborate with one PACT teamlet vs. a larger number of PACT staff). The TDM will be administered at baseline, 6 and 12 months via an email link to an online survey portal.

In addition to being included in the project’s evaluation, the results of both measures, along with available normative data, will be used to support the facilitated implementation process. To do this, external facilitators will share the results of the site data and use ORC and TDM normative data to review the results with the PACT teams, pinpoint areas for improvement, and identify realistic action steps to improve team development and functioning in service of supporting the integration of PSs into the PACT team.

Qualitative interviews to assess key barriers and facilitators to integrating a PS into PACT will be developed based on i-PARIHS [51], with questions derived from generic items in the i-PARIHS Guide [68]. These interviews will be conducted with PSs and at least one other PACT staff (usually the PS supervisor) at each site near the end of facilitation. Through these 60–90-min telephone interviews (*n* = 60), we will capture i-PARIHS’ core elements of evidence, context, and facilitation. For the sites receiving facilitated implementation, interviews will include additional questions about that experience (e.g., if and how they worked with the external facilitator, what it was like, if it helped, what could have been better). Interviews will be digitally audio-recorded and transcribed.

#### Service delivery measures

Peer Workload. Services provided by each PS involved in the project will be collected. Both the number of unique Veterans who receive PS services of those who are eligible (*R*each) and the number of contacts provided by the PS at each site (*A*doption) will be assessed. This data will be obtained for all sites via biweekly data pulls from the VA Corporate Data Warehouse (CDW) conducted by staff of the Veterans Engineering Resource Center (VERC) at the VA Pittsburgh Healthcare System. VERC staff will develop a custom database from data stored in the CDW utilizing SQL routines that can be updated to include new project sites or PSs.

The Peer Fidelity Measure [69] is designed to assess the services provided by PSs and factors that support and hamper the performance of those services (*I*mplementation). The measure addresses two broad domains: (a) the degree to which a PS provides 16 different services shown to be critical for PSs according to the literature and expert panel (e.g., serving as a role model for recovery; sharing recovery story; increasing client’s participation in own illness management) and (b) the degree to which 16 different factors are having a positive or negative influence on PS implementation (e.g., well understood expectations for PS work, support for PS at higher organizational levels, regular supervision, access to education and training). Each service and implementation factor has one to two questions that asks about their presence (responses ranging from 1 = not at all to 5 = very much). Although the measure is in early stages of testing, preliminary results from a small pilot test showed the measure of PS services may distinguish between PSs with fewer and greater years of experience [69]. Results from this project will yield data that will be used to further test its psychometric properties. There are separate versions for PSs, their supervisors, and patients, but the items overlap in content. To allow time for the PS to establish their services, each PS and their supervisor will complete the measure 6 months after the first service provided and again 6 months later (again, by email link to an online portal). All Veterans with whom each PS works will be asked to complete the measure at the 6- and 12-month survey timepoints along with the outcome measures described below.

#### Veteran outcome measures

Veterans receiving PS services will be asked to complete measures of activation, satisfaction and health status, in addition to age, gender, race/ethnicity, service connection, medical diagnosis, and contact information. At baseline (before the first PS service contact), the PS will collect the measures in person. At 6 and 12 months (from first PS service contact), project staff will collect the measures by phone.

The Satisfaction Index-Mental Health [70] is a 12-item, unidimensional measure shown to be valid, reliable, and sensitive to change in a sample of Veterans with mental illnesses being treated in primary care settings. Six additional questions taken from VHA’s annual Survey of Health Experience of Veterans (SHEP) will be added, including two Yes/No items: talked with your provider about specific health goals, provider talked about what makes it hard to take care of health (yes/no); and four 5-point items (strongly agree to strongly disagree): got service I needed, was easy to get the service I needed, felt like a valued customer, trust VA to fulfill our country’s commitment to Veterans.

The Patient Activation Measure (PAM) is a 13-item survey that measures an individual’s perceived ability to manage his or her illness and health behaviors and act as an effective patient. It has been shown to be reliable, valid, sensitive to change, and correlate with measures of improved self-management and health outcomes [31–33].

One item from the *VR-12*, a Veteran version of the SF-36 Health Inventory [71–73], will be used to assess general health. The item, “In general, would you say your health is…” asks Veterans to respond on a 5-point scale ranging from excellent to poor. The use of this single item is backed up by large body of research in which the item is associated with specific health problems, mortality, use of health services, changes in functioning, and recovery from poor health [74].

### Data analysis

The overall qualitative analysis strategy is described here. Additional qualitative analysis description and the statistical analyses are described under each hypothesis below. Transcripts of the qualitative interviews will be analyzed using rapid assessment, a team-based, iterative data collection and analysis approach [75]. A transcript summary template with domains based on the elements of i-PARIHS and other key topics (e.g., degree of PS’s integration into the PACT, the experience of facilitated implementation) will be collaboratively developed by the evaluation team. After testing and revising the template with at least the first five transcripts, a finalized version will be developed. Thereafter, evaluation team members will independently use the summary template to closely read each transcript and summarize content under each domain, with periodic checks for consensus and reliability. The use of a template will facilitate the rapid but thorough review and reduction of data from a large number of interviews, enabling the comparison and identification of themes both within and across sites, study conditions (standard or facilitated implementation), and stakeholder roles (PS or supervisor) [70]. Furthermore, because preliminary analysis will begin as soon as the first transcripts are available, this approach will allow for iterative changes to the interview guides as new questions arise from the analysis.

#### Hypothesis 1

After 1 year, sites in the facilitated implementation group will demonstrate better Reach, Adoption, and Implementation than sites in the standard implementation group.

Reach will be operationalized as the number of unique patients seen by a PS, per hour of time a PS works per month at the site. Sites vary in the number of hours per week PSs are able to work (because sites were receiving no additional funds to support the PSs, they had to use existing PSs). Poisson regression will be used to model the counts of unique patients seen per month over the 12-month implementation period. The number of hours per month the PS was working will be used as an offset to control for the different number of hours worked by each PS. A generalized linear mixed model will be used to allow for missing data. Site and time will be included in the model as random variables with intervention condition as a fixed effect. The z-test of the time by intervention condition interaction will be the test of the hypothesis. The quantitative assessment of Reach will be further enhanced by information from the qualitative interviews, including descriptions of PSs’ caseload and patterns of interaction with Veteran patients (e.g., in-person meetings, follow-up phone calls, group setting contacts).

Adoption will be operationalized as the number PS services (contacts) delivered, per hour of time a PS works per month. PSs are dependent on primary care staff for referrals, thus this measure of service delivery reflects the extent to which a site has fully adopted the PS role. The same model described for Reach will be used for the analysis of adoption using total number of contacts as the dependent variable. Analysis of the qualitative interviews, focusing on discussions of the PSs’ level of integration into PACT, how they receive referrals, and their relationships with PACT co-workers, will add to our quantitative analysis of Adoption.

Implementation will be operationalized as the six scores from the Peer Fidelity Measure: Peer services (PS-rated, supervisor-rated, and Veteran-rated) and Implementation factors (PS-rated, supervisor-rated, and Veteran-rated). A separate analysis will be performed for each of the six dependent measures at each timepoint. A general linear mixed model will be used to compare the two intervention condition of each dependent measure. For the PS-rated and Veteran-rated outcomes, site will be included as a random effect. Information learned from the qualitative interviews will be used to triangulate the data from the quantitative measures of Implementation. Specifically, information regarding the context, the role of the PS in PACT, and what the PS adds to PACT services will be analyzed.

#### Hypothesis 2

Veterans served by PSs in the facilitated implementation group will demonstrate greater improvements over time in satisfaction, activation, and functioning (Effectiveness) than Veterans served by PSs in the standard implementation group.

The data for hypothesis 2 will be analyzed with a comparison of Veterans’ mean scores (satisfaction, activation, and functioning) using a mixed effects model with random intercept and slope (standard growth curve model) and interaction term between evaluation condition and time. Veteran demographic covariates (age, gender, race/ethnicity, service connection, and ICD9 diagnosis codes) will be included in the mixed effect models to control for differential outcomes and to identify significant demographic factors influencing Effectiveness. We will further model the impact of various aspects of PS service delivery (ORC and TDM scores, absolute value of services delivered, rate of services delivered, caseload size, etc.) on the patient outcomes of satisfaction, activation, and functioning. Across outcomes, we estimate we will have greater than 89% power to detect a small effect (*d* = .3) between intervention groups at 1 year, with intracluster correlation = .025, an alpha level = .05 (two-tailed), and controlling for baseline. We estimate that each PS will see at least 14 Veterans per month for 84 Veterans per PS. Assuming a conservative 50% attrition at 1 year and 70% at 2 years, we will have a sample of 2100, 1050, 630 at baseline, 1 year, and 2 years, respectively. With the same assumptions, we will have 83% power at year 2 to detect the same size effect.

#### Hypothesis 3

In the second year (1 year after the end of facilitation), the facilitated implementation group will continue to demonstrate superior Maintenance (performance on measures of Reach, Adoption, and Implementation). The analyses for Reach, Adoption, and Implementation conducted after 1 year will be repeated in the second year.

### Trial status

The project was deemed a Quality Improvement project on 2/24/2016. This determination was made after two actions. First, on 12/3/2015, the PIs (MC, RG) completed a non-research determination checklist that asked several questions about the nature of the project (e.g., yield generalizable knowledge, expanding knowledge base, double-blind or placebo design). All answers indicated the project was not a research study. We sent the completed checklist to the VA’s Chief Consultant of Mental Health Services to obtain final approval. In addition, we obtained approval on 3/10/2016 from Organizational Assessment Sub-Committee (OASC) of the Office of Research Development, which governs the surveying of VA staff (peer specialists are VA staff).

Site recruitment began in August 2015 and is ongoing. Cohort 1 (*n* = 8) sites were paired and randomized in December 2015. Official start date for cohort 1 was February 1, 2016. One control site dropped out in January 2016, following randomization and prior to initiation of facilitation, leaving seven sites in cohort 1. Data collection began on March 2016 and is ongoing. Veterans were provided with information about the project in a disclosure statement that had the basic elements of an informed consent. Cohort 2 (*n* = 10) was randomized and officially began on July 2016. Because staff resources were not sufficient to provide facilitated implementation to five sites, after the initial matching of sites, one pair was randomly chosen and removed from the group assignment procedure, with both sites in that pair receiving standard implementation. The remaining four pairs were randomized, resulting in cohort 2 (*n* = 6) and facilitated implementation (*n* = 4) groups. Cohort 3 is scheduled to start in March–April of 2017.

## Discussion

As noted above, research evidence and passive dissemination strategies often do not change clinical practice or increase the adoption of new practices. PS services has some demonstrated evidence, yet has not been widely adopted among VHA PACT. This project intends to test whether a comprehensive implementation strategy—i-PARIHS based facilitation tailored to PS deployment—can aid in PS implementation in PACT, and secondarily to evaluate the effectiveness of PS services on Veteran activation, satisfaction, and functioning. Further, this project will yield important lessons about how and under what conditions facilitation aids PS implementation. The test will be challenging as PACT are under significant pressure to serve a large number of Veterans. However, the PACT randomized to receive facilitation will have a number of additional supports to assist with the implementation of PS services. If successful, this type of facilitation could be used across a larger number of PACT as VHA considers expanding the deployment of PSs in primary care. In addition, study results will continue to evaluate the efficacy of PS for the helping Veterans address a number of preventable chronic health conditions.

This project is registered at ClinicalTrials.gov with number NCT02732600 (URL:https://clinicaltrials.gov/ct2/show/NCT02732600). The trial was first registered April 4 2016.

## Abbreviations

CDSMP: Chronic Disease Self-Management Program
i-PARIHS: Integrated Promoting Action Research on Implementation in Health Services
ORC: Organizational Readiness for Change
PACT: Patient Aligned Care Teams
PSs: Peer specialists
QUERI: Quality Enhancement Research Initiative
TDM: Team Development Measure
VAMC: Veterans Affairs Medical Centers
VHA: Veterans Health Administration
